# Intestinal microbe-dependent ω3 lipid metabolite αKetoA prevents inflammatory diseases in mice and cynomolgus macaques

**DOI:** 10.1038/s41385-021-00477-5

**Published:** 2022-01-10

**Authors:** Takahiro Nagatake, Shigenobu Kishino, Emiko Urano, Haruka Murakami, Nahoko Kitamura, Kana Konishi, Harumi Ohno, Prabha Tiwari, Sakiko Morimoto, Eri Node, Jun Adachi, Yuichi Abe, Junko Isoyama, Kento Sawane, Tetsuya Honda, Asuka Inoue, Akiharu Uwamizu, Takashi Matsuzaka, Yoichi Miyamoto, So-ichiro Hirata, Azusa Saika, Yuki Shibata, Koji Hosomi, Ayu Matsunaga, Hitoshi Shimano, Makoto Arita, Junken Aoki, Masahiro Oka, Akira Matsutani, Takeshi Tomonaga, Kenji Kabashima, Motohiko Miyachi, Yasuhiro Yasutomi, Jun Ogawa, Jun Kunisawa

**Affiliations:** 1 Laboratory of Vaccine Materials, Center for Vaccine and Adjuvant Research and Laboratory of Gut Environmental System, National Institutes of Biomedical Innovation, Health and Nutrition (NIBIOHN), 7-6-8 Asagi Saito, Ibaraki, Osaka, 567-0085 Japan; 2 Division of Applied Life Sciences, Graduate School of Agriculture, Kyoto University, Kitashirakawa-oiwakecho, Sakyo-ku, Kyoto, 606-8502 Japan; 3 Laboratory of Immunoregulation and Vaccine Research, Tsukuba Primate Research Center, NIBIOHN, 1-1 Hachimandai, Tsukuba, Ibaraki, 305-0843 Japan; 4 Department of Physical Activity Research, NIBIOHN, 1-23-1 Toyama, Shinjuku-ku, Tokyo, 162-8636 Japan; 5 Laboratory of Proteome Research and Laboratory of Proteomics for Drug Discovery, NIBIOHN, 7-6-8 Asagi Saito, Ibaraki, Osaka, 567-0085 Japan; 6 Division of Molecular Diagnostics, Aichi Cancer Center Research Institute, 1-1 Kanokoden, Chikusa-ku, Nagoya, 464-8681 Japan; 7 Graduate School of Pharmaceutical Sciences, Osaka University, 1-1 Yamadaoka, Suita, Osaka, 565-0871 Japan; 8 Department of Dermatology, Kyoto University Graduate School of Medicine, 54 Shogoin Kawara-cho, Kyoto, 606-8507 Japan; 9 Department of Dermatology, Hamamatsu University School of Medicine, 1-20-1 Handayama, Higashi-ku, Hamamatsu, Shizuoka, 431-3192 Japan; 10 Department of Molecular and Cellular Biochemistry, Graduate School of Pharmaceutical Sciences, Tohoku University, 6-3 Aoba, Aramaki, Aoba-ku, Sendai, Miyagi 980-8578 Japan; 11 Graduate School of Pharmaceutical Sciences, The University of Tokyo, 7-3-1 Hongo, Bunkyo-ku, Tokyo, 113-0033 Japan; 12 Department of Endocrinology and Metabolism, Faculty of Medicine, University of Tsukuba, 1-1-1 Tennodai, Tsukuba, Ibaraki, 305-8575 Japan; 13 Transborder Medical Research Center, University of Tsukuba, 1-1-1 Tennodai, Tsukuba, Ibaraki, 305-8575 Japan; 14 Laboratory of Nuclear Transport Dynamics, NIBIOHN, 7-6-8 Asagi Saito, Ibaraki, Osaka, 567-0085 Japan; 15 Department of Microbiology and Immunology, Kobe University Graduate School of Medicine, 7-5-1 Kusunoki-cho, Chuo-ku, Kobe, Hyogo, 650-0017 Japan; 16 Faculty of Agriculture, Takasaki University of Health and Welfare, 54 Nakaoruimachi, Takasaki, Gumma 370-0033 Japan; 17 Division of Physiological Chemistry and Metabolism, Keio University Faculty of Pharmacy, 1-5-30 Shibakouen, Minato-ku, Tokyo, 105-8512 Japan; 18 Laboratory for Metabolomics, RIKEN Center for Integrative Medical Sciences, 1-7-22 Suehiro-cho, Tsurumi-ku, Yokohama, Kanagawa 230-0045 Japan; 19 Cellular and Molecular Epigenetics Laboratory, Graduate School of Medical Life Science, Yokohama City University, 1-7-29 Suehiro-cho, Tsurumi-ku, Yokohama, Kanagawa 230-0045 Japan; 20 Department of Internal Medicine, Shunan City Shin-nanyo Hospital, 2-3-15 Miyanomae, Shunan, Yamaguchi, 746-0017 Japan; 21 International Research and Development Center for Mucosal Vaccines, The Institute of Medical Science, The University of Tokyo, 4-6-1 Shirokanedai, Minato-ku, Tokyo, 108-8639 Japan; 22 Graduate School of Medicine, Graduate School of Dentistry, Osaka University, 1-1 Yamadaoka, Suita, Osaka, 565-0871 Japan; 23 Research Organization for Nano and Life Innovation, Waseda University, Tokyo, 162-0041 Japan

## Abstract

Dietary ω3 fatty acids have important health benefits and exert their potent bioactivity through conversion to lipid mediators. Here, we demonstrate that microbiota play an essential role in the body’s use of dietary lipids for the control of inflammatory diseases. We found that amounts of 10-hydroxy-*cis*-12-*cis*-15-octadecadienoic acid (αHYA) and 10-oxo-*cis*-12-*cis*-15-octadecadienoic acid (αKetoA) increased in the feces and serum of specific-pathogen-free, but not germ-free, mice when they were maintained on a linseed oil diet, which is high in α-linolenic acid. Intake of αKetoA, but not αHYA, exerted anti-inflammatory properties through a peroxisome proliferator-activated receptor (PPAR)γ-dependent pathway and ameliorated hapten-induced contact hypersensitivity by inhibiting the development of inducible skin-associated lymphoid tissue through suppression of chemokine secretion from macrophages and inhibition of NF-κB activation in mice and cynomolgus macaques. Administering αKetoA also improved diabetic glucose intolerance by inhibiting adipose tissue inflammation and fibrosis through decreased macrophage infiltration in adipose tissues and altering macrophage M1/M2 polarization in mice fed a high-fat diet. These results collectively indicate that αKetoA is a novel postbiotic derived from α-linolenic acid, which controls macrophage-associated inflammatory diseases and may have potential for developing therapeutic drugs as well as probiotic food products.

## Introduction

The incidence of allergic and inflammatory skin diseases and metabolic disorders, including type 2 diabetes, is increasing.^1–3^ Accumulating evidence suggests that quantity of dietary lipid is a critical determinant in the development of inflammatory diseases.^4,5^ In addition to the quantity of dietary lipids, their fatty acid composition plays important roles in the regulation of inflammatory diseases. In fact, the potential benefits of ω3 fatty acids in prevention of inflammatory vascular disease were discovered in a cohort study more than 40 years ago.^6^ Yet, the beneficial effects of ω3 fatty acids in clinical studies remain debated.^7–9^

Recent evidence suggests that the metabolism of dietary ω3 fatty acids is a key factor which influences their effectiveness in the regulation of health and diseases. The conversion of ω3 fatty acids into bioactive metabolites is mediated by mammalian enzymes including cyclooxygenase (COX), lipoxygenase (LOX), and cytochrome P450 (CYP).^10,11^ Eicosapentaenoic acid (EPA), n-3 docosapentaenoic acid (DPA), and docosahexaenoic acid (DHA) are representative ω3 fatty acids, which exert pro-resolution and anti-inflammatory properties through their conversion into bioactive lipid mediators, including EPA-derived resolvins, 17,18-epoxyeicosatetraenoic acid (EpETE) and 15-hydroxyeicosapentaenoic acid, n-3 DPA-derived 14-hydroxy DPA, and DHA-derived protectins and maresins.^12–18^ These studies highlight that conversion of ω3 fatty acids into bioactive metabolites is essential for the regulatory roles of these lipids.

In addition, intestinal bacteria contribute to dietary lipid metabolism and produce unique, non-mammalian lipid metabolites with potent biologic activities.^19–21^ For example, *Lactobacillus plantarum* AKU1009a use saturation metabolism by bacterial CLA-HY enzyme to convert ω6 linoleic acid to 10-hydroxy-*cis*-12-octadecenoic acid (HYA).^19^ HYA is further converted to 10-oxo-*cis*-12-octadecenoic acid (KetoA) by bacterial CLA-DH enzyme.^19^ These metabolites exert potent biologic activities.^22–27^ In addition, ω3 α-linolenic acid is reportedly metabolized in *L. plantarum* AKU1009a, too, with both the ω3 and ω6 forms undergoing the same transformations. α-Linolenic acid is metabolized to 10-hydroxy-*cis*-12-*cis*-15-octadecadienoic acid (αHYA) and 10-oxo-*cis*-12-*cis*-15-octadecadienoic acid (αKetoA) by CLA-HY and CLA-DH found in *L. plantarum* AKU1009a.^20,28,29^ However, the biologic activities of αHYA and αKetoA remain unclear.

In this study, we found that αKetoA exerted potent anti-inflammatory activities for the control of contact hypersensitivity in both mice and non-human primates and for the amelioration of diabetes in mice fed a high-fat diet (HFD) through regulating macrophage activity in a peroxisome proliferator-activated receptor (PPAR)γ-dependent manner. These results extend our knowledge by revealing the important roles of bacteria in accomplishing the health-promoting effects of ω3 fatty acids by generating the unique intestinal microbial lipid metabolite αKetoA.

## Results

### αKetoA and αHYA are ω3 α-linolenic acid-derived and intestinal bacteria-dependent lipid metabolites

**Fig. 1:**
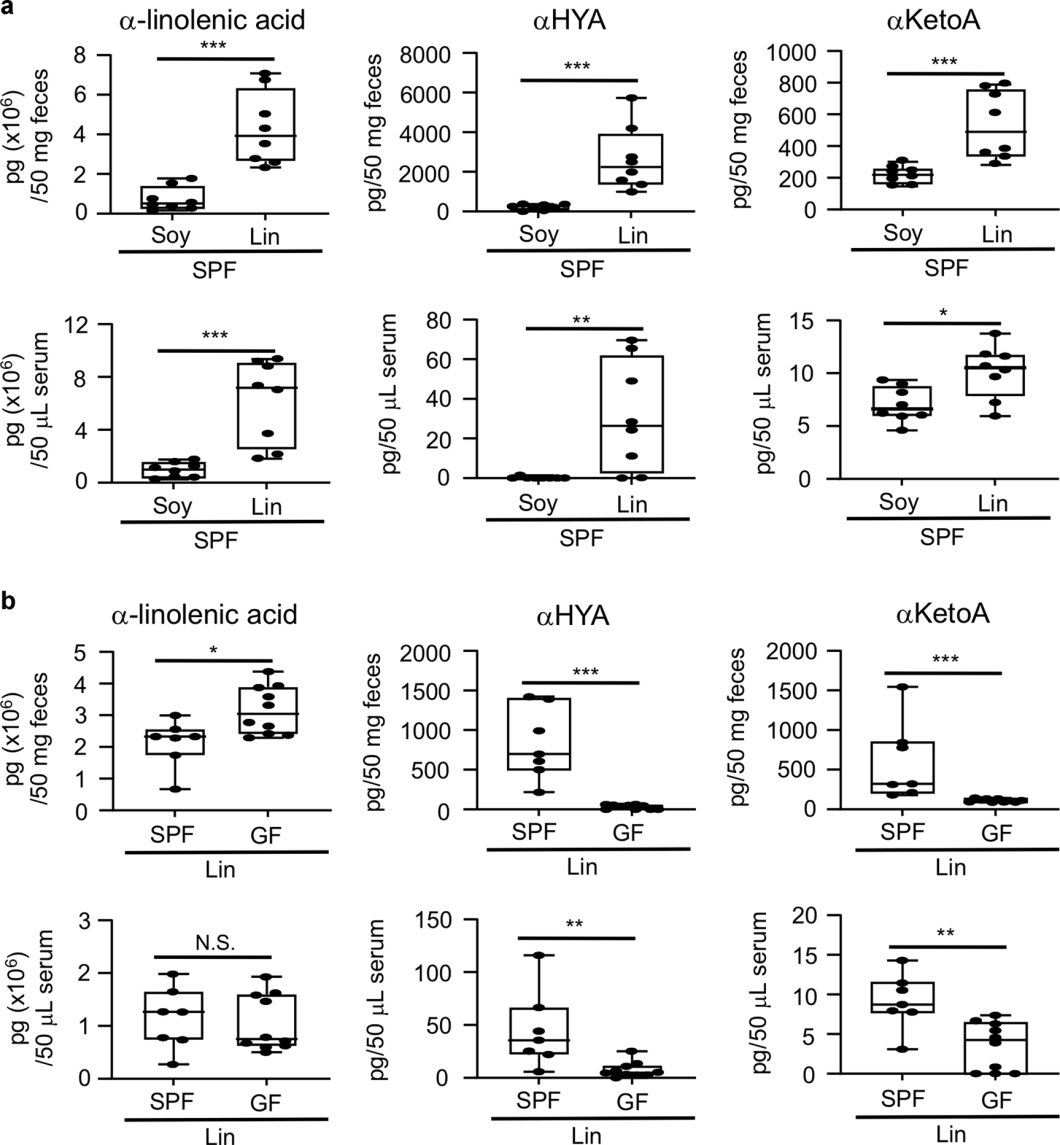
αHYA and αKetoA are ω3 fatty acid- and intestinal bacteria-dependent metabolites. **a** Mice were fed a chemically defined diet containing either soybean oil or linseed oil under SPF housing conditions for 2 months, and the amounts of fatty acids in the feces and serum were analyzed through LC-MS/MS. **b** Mice were fed a chemically defined diet containing linseed oil under either SPF or GF housing conditions for 2 months, after which the amounts of fatty acids in the feces and serum were analyzed through LC-MS/MS. Each point represents data from individual mice. Statistical significance was evaluated by using the Mann–Whitney test; ***p < 0.001; **p < 0.01; *p < 0.05; N.S. not significant.

We first sought to examine whether dietary intake of linseed oil, which is high in α-linolenic acid, increases the amount of αHYA and αKetoA in mouse feces. We fed mice a diet containing either soybean oil (Soy-mice) or linseed oil (Lin-mice) for 2 months and collected feces for the analysis of fatty acid metabolites. Consistent with the fatty acid composition of the dietary oils, lipidomic analysis through liquid chromatography–tandem mass spectrometry (LC-MS/MS) revealed that the amount of α-linolenic acid was higher in the feces of Lin-mice than in those of Soy-mice (Fig. 1a). We also found that the amounts of αHYA and αKetoA were increased in the feces of Lin-mice (Fig. 1a). Furthermore, Lin-mice also showed increased serum levels of α-linolenic acid, αHYA, and αKetoA (Fig. 1a).

We next fed a linseed oil-containing diet to mice maintained under either specific-pathogen-free (SPF) or germ-free (GF) housing conditions for 2 months. The lipidomic analysis revealed that the amount of α-linolenic acid in the serum was comparable between SPF and GF mice (Fig. 1b). In contrast, the amounts of αHYA and αKetoA were lower or absent in the feces and serum of GF mice than in those of SPF mice (Fig. 1b). These results demonstrate that αHYA and αKetoA are lipid metabolites derived from ω3 α-linolenic acid and that their generation in the intestine and subsequent absorption into the body is dependent on the presence of intestinal bacteria.

### Contact hypersensitivity is ameliorated by αKetoA through PPARγ-dependent inhibition of the development of inducible skin-associated lymphoid tissue (iSALT)

**Fig. 2:**
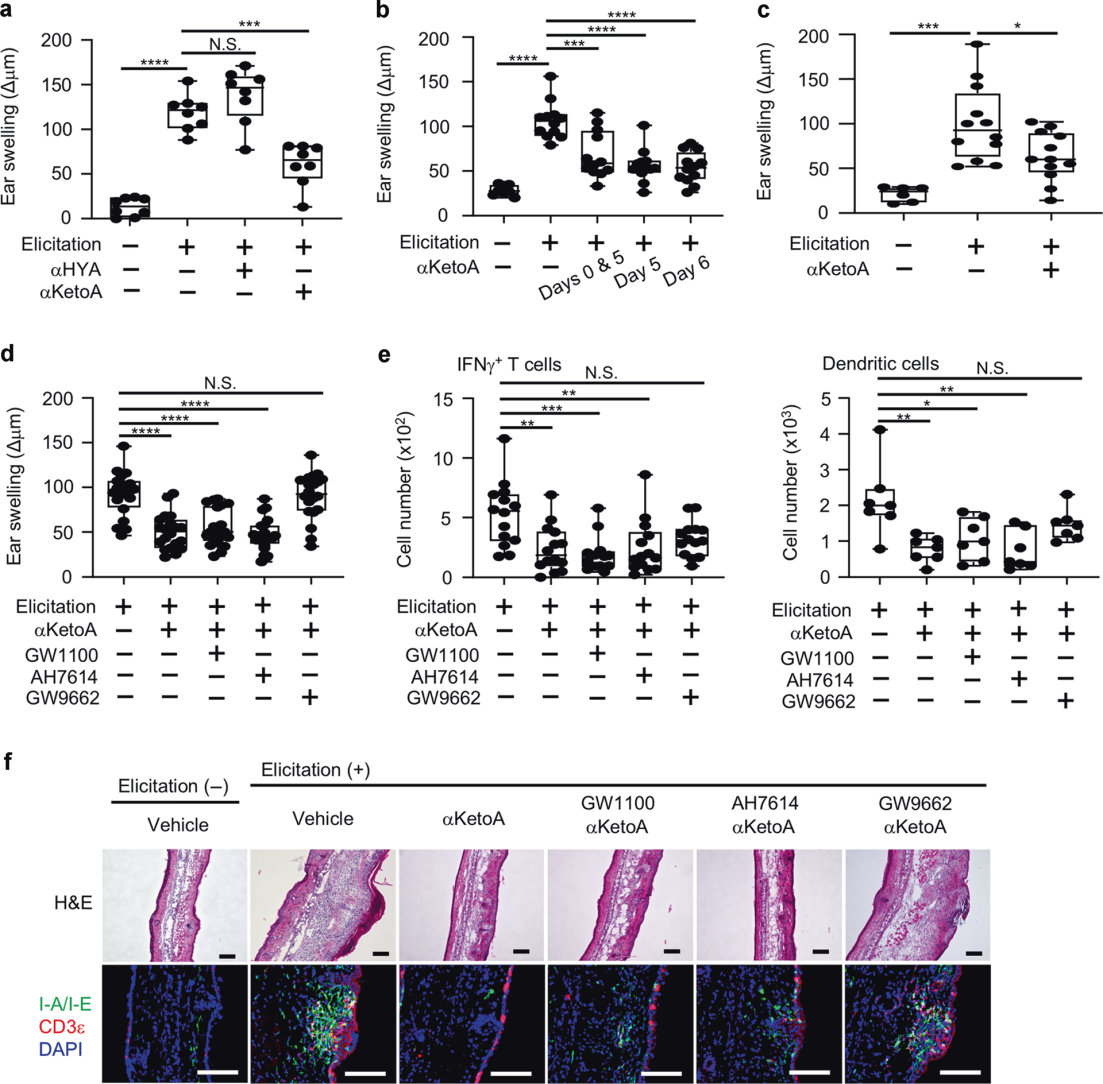
Contact hypersensitivity is ameliorated by αKetoA, but not αHYA, through PPARγ-dependent inhibition of iSALT development. **a** Mice orally received αHYA (dose: 1 μg/mouse), αKetoA (dose: 1 μg/mouse), or 0.5% (vol/vol) ethanol dissolved in PBS (vehicle control) on days −10 to −6, days −3 to 1, and days 4−6; DNFB-induced ear swelling was evaluated on day 7. Data are combined from two independent experiments. **b** Mice orally received αKetoA (dose: 10 μg/mouse) or 0.5% (vol/vol) ethanol dissolved in PBS (vehicle control) on the days indicated 90 min before DNFB stimulation on days 0 and 5; DNFB-induced ear swelling was evaluated on day 7. Data are combined from two independent experiments. **c** Mice were topically treated with αKetoA (dose: 10 μg/mouse) or 50% (vol/vol) ethanol dissolved in PBS (vehicle control), 30 min before DNFB stimulation on days 0 and 5, and DNFB-induced ear swelling was evaluated on day 7. Data are combined from two independent experiments. **d** Mice were intraperitoneally injected with either GW1100, AH7614, or GW9662 30 min before oral administration of αKetoA on days 0 and 5. Mice were challenged with DNFB 90 min after oral administration of αKetoA, and ear swelling was evaluated on day 6. Data are combined from three independent experiments. **e** Mice were intraperitoneally injected with either GW1100, AH7614, or GW9662 30 min before oral administration of αKetoA on days 0 and 5. Mice were challenged with DNFB 90 min after oral administration of αKetoA, and the numbers of IFNγ^+^ T cells (7-AAD^−^CD45^+^TCRβ^+^IFNγ^+^) and dendritic cells (7-AAD^−^CD45^+^CD11c^+^F4/80^–^I-A^b+^CD11b^+^) were calculated on the basis of total cell numbers and flow cytometric data on days 6 and 7, respectively. Data of IFNγ^+^ T cells and dendritic cells are combined from 4 and 2 independent experiments, respectively. **f** Mice were intraperitoneally injected with either GW1100, AH7614, or GW9662 30 min before oral administration of αKetoA on days 0 and 5. Mice were challenged with DNFB 90 min after the oral administration of αKetoA. Ears were obtained on day 7 and frozen sections were stained with hematoxylin and eosin or the indicated antibodies and reagent. Elicitation (-) indicates mice that were not stimulated with DNFB on day 5 and used as a control. Data are representative of three independent experiments. Scale bars, 100 μm. Each point represents data from individual mice Statistical significance was evaluated by using one-way ANOVA; ****p < 0.0001; ***p < 0.001; ** *p* < 0.01; * *p* < 0.05; N.S. not significant.

We then examined whether αHYA and αKetoA exert anti-inflammatory properties. To address this issue, we applied the 2,4-dinitrofluorobenzene (DNFB)-induced murine contact hypersensitivity model, a representative type IV skin allergic inflammation model that comprises sensitization and elicitation phases. In the sensitization phase, skin exposure to DNFB activates skin dendritic cells to migrate to the regional lymph nodes and activate naive T cells and consequently induce Th1 and Tc1 cells.^30^ In the elicitation phase, re-exposure to DNFB induces the development of iSALT, which enhances the production of IFNγ by skin effector T cells in situ.^30^ We orally administered the fatty acid metabolite to mice from before sensitization and during the experimental protocol, and we evaluated ear swelling as a representative inflammatory sign of contact hypersensitivity. We found that DNFB-induced ear swelling was ameliorated by oral administration of αKetoA but not αHYA (Fig. 2a). We next examined the therapeutic effects of αKetoA by administering it orally to mice at 1 day after elicitation with DNFB and measuring ear swelling the day after αKetoA administration. We found that αKetoA treatment effectively ameliorated ear swelling (Fig. 2b). Topical treatment with αKetoA also exerted anti-inflammatory activity in the inhibition of ear swelling (Fig. 2c).

We next sought to examine molecular mechanisms of αKetoA in the amelioration of contact hypersensitivity. The carbon length of fatty acids is an important determinant of receptor specificity: short-chain fatty acids are recognized by GPR41 and GPR43, whereas long-chain fatty acids are recognized by GPR40 and GPR120.^31^ In addition, long-chain fatty acids are directly recognized by PPARγ.^32^ To identify the functional receptor of αKetoA, we applied specific antagonist treatment in the contact hypersensitivity model, using GW1100, AH7614, and GW9662 as selective antagonists of GPR40, GPR120, and PPARγ, respectively. We found that the anti-inflammatory effect of αKetoA was dependent on PPARγ but not GPR40 or GPR120, according to ear swelling at 24 h after elicitation (Fig. 2d). This effect continued 48 h after the elicitation (Supplementary Fig. S1). Consistent with the independence of αKetoA from GPR40 and GPR120 in exerting anti-inflammatory activity, transforming growth factor (TGF)α-shedding assays revealed that αKetoA had little activity in inducing GPR40- and GPR120-mediated signaling when compared with the positive control, 13-oxo-*cis*-9,*cis*-15-octadecadienoic acid (Supplementary Fig. S2).^33^

We next sought to examine cellular dynamics in the treatment with αKetoA. Flow cytometry analysis revealed that αKetoA decreased the number of IFNγ^+^ T cells and dendritic cells in the ear skin in a PPARγ-dependent manner (Fig. 2e). Histologic analysis revealed that αKetoA disrupted the iSALT structure; this disruption was dependent on PPARγ but independent from GPR40 and GPR120 (Fig. 2f). These results indicate that the αKetoA–PPARγ axis ameliorates contact hypersensitivity by inhibiting IFNγ production by T cells through the disruption of iSALT formation.

We have developed a hapten-induced contact hypersensitivity model in cynomolgus macaques.^13^ In this model, macaques are sensitized with DNFB on the abdominal skin and then stimulated with DNFB on the back, thus inducing skin inflammatory signs, including epidermal hyperplasia and inflammatory cell infiltration (Supplementary Fig. S3).^13^ In the current study, we used this model to address whether αKetoA exerts anti-inflammatory activity in non-human primates and found that, as in the murine model, DNFB-induced skin inflammatory signs such as epidermal hyperplasia and the accumulation of CD3^+^ T cells were inhibited by topical application of αKetoA (Supplementary Fig. S3). These results show that αKetoA is effective for the treatment of skin inflammation not only in rodents but also in non-human primates and therefore is a promising candidate for drug development.

### αKetoA inhibited chemokine expression by interfering with nuclear translocation of NF-kB in macrophages

**Fig. 3:**
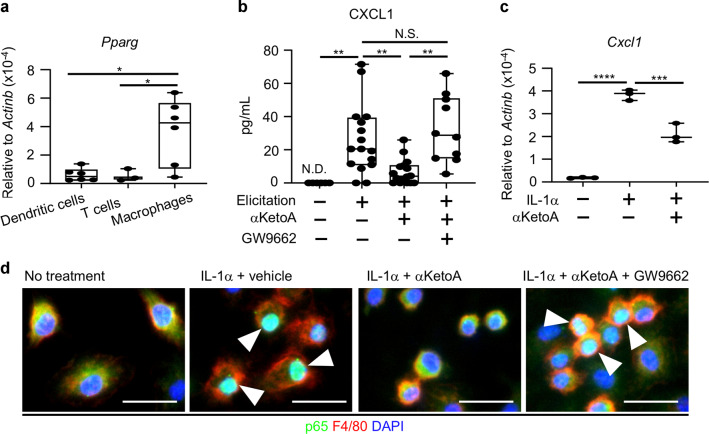
αKetoA inhibits chemokine expression in macrophages by interfering with the nuclear translocation of NF-κB. **a** Dendritic cells (7-AAD^−^CD45^+^CD11c^+^Gr1^−^F4/80^−^) and macrophages (7-AAD^−^CD45^+^Gr1^Low^CD11b^+^) were isolated from mouse ear skin on day 7 of the contact hypersensitivity model, and the gene expression level of *Pparg* was measured through reverse transcription and quantitative PCR analysis and normalized to that of *Actinb*. Data are combined from two independent experiments. **b** Ear homogenates were prepared on day 6 and 7 of the contact hypersensitivity model and examined by ELISA to determine the amount of CXCL1. Data are combined from five independent experiments. **c**, **d** In vitro assay of bone marrow-derived macrophages. Bone marrow cells were incubated as described in the Methods section and stimulated with IL-1α with or without αKetoA to examine the gene expression level of *Cxcl1* (**c**) and nuclear translocation of NF-κB (**d**). **c** The gene expression levels were normalized to that of *Actinb*. Data are representative of three independent experiments with similar results (triplicate assay). **d** NF-κB and macrophages were visualized by staining with anti-p65 mAb (green) and anti-F4/80 mAb (red), respectively; nuclei were stained with DAPI (blue). Nuclear translocation of p65 is indicated as a change in the color of the nucleus to turquoise (arrowheads). Data are representative of three independent experiments. Scale bars, 20 μm. Each point represents data from individual mice. Statistical significance was evaluated by using one-way ANOVA; ****p < 0.0001; ***p < 0.001; **p < 0.01; *p < 0.05; N.S. not significant, N.D. not detected.

To identify the target cells of αKetoA, we compared the gene expression level of *Pparg* among dendritic cells, T cells, and macrophages in the skin; dendritic cells and T cells are essential constituents of iSALT, and macrophages act as iSALT inducer cells.^34^ We found that macrophages expressed the highest level of *Pparg* among these cells (Fig. 3a). This finding prompted us to examine whether αKetoA affected the expression of macrophage-derived chemokines that recruit CXCR2^+^ dendritic cells for the formation of iSALT.^34^ Treatment of mice with αKetoA reduced the amount of CXCL1 in ear homogenates, with minimal effects on CXCL2 levels (Fig. 3b and Supplementary Fig. S4a). In addition, treatment with GW9662 abrogated the effects of αKetoA on CXCL1 levels, thus indicating their dependency on PPARγ (Fig. 3b and Supplementary Fig. S4a).

We then prepared bone marrow-derived macrophages and stimulated them with IL-1α to induce CXCR2 ligands.^34^ We found that αKetoA inhibited IL-1α-mediated induction of *Cxcl1* and *Cxcl2* (Fig. 3c and Supplementary Fig. S4b). To induce *Cxcl1* and other pro-inflammatory cytokines and chemokines, the signaling pathway from IL-1α activates NF-κB,^35^ therefore we asked whether αKetoA inhibited the nuclear translocation of NF-κB, which is an essential step for NF-κB-mediated gene expression. We found that αKetoA inhibited IL-1α-mediated nuclear translocation of NF-κB in a PPARγ-dependent manner (Fig. 3d). These results collectively indicate that the αKetoA–PPARγ axis ameliorated contact hypersensitivity by disrupting iSALT formation through inhibiting NF-κB activation and chemokine expression in macrophages.

### HFD-induced glucose intolerance is ameliorated by αKetoA through inhibiting adipose tissue inflammation

Given that αKetoA targets macrophages, we examined whether αKetoA ameliorates other macrophage-associated inflammatory diseases. Several lines of evidence indicate that obesity is associated with adipose tissue inflammation due to recruitment of pro-inflammatory M1 macrophages and contributes to the development of metabolic disorders, including diabetic glucose intolerance.^36–38^ When we fed mice an HFD combined with oral administration of either αKetoA or vehicle (as a control) for several months, neither body weight increase nor the weight of the epididymal adipose tissue differed between the 2 groups, suggesting that αKetoA did not affect the development of obesity (Supplementary Fig. S5a–c).

**Fig. 4:**
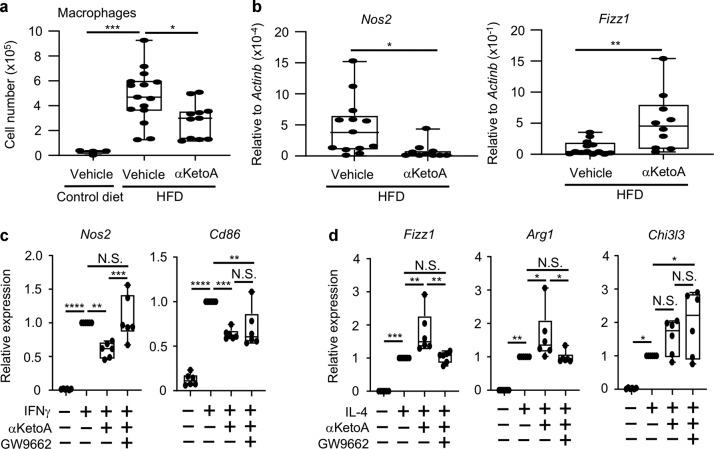
αKetoA induces M2 macrophage polarization in the adipose tissue of HFD mice. **a** Mice were fed either a control diet containing soybean oil or HFD for 3 months with or without oral administration of αKetoA (dose: 10 μg/mouse, 3 times/week), and epididymal adipose tissues were analyzed through flow cytometry. The number of macrophages (7-AAD^−^CD45^+^Ly6G^−^F4/80^+^CD11b^+^) was calculated on the basis of total cell numbers and flow cytometric data. Data are combined from four independent experiments. **b** After the HFD was fed for 4 months with or without oral administration of αKetoA (dose: 10 μg/mouse, 3 times/week), macrophages were isolated from epididymal adipose tissues and examined for gene expression of *Nos2* and *Fizz1* as markers of M1 and M2 macrophages, respectively. Data are combined from four independent experiments. **c**, **d** In vitro assay of bone marrow-derived macrophages. Gene expression levels were normalized to that of *Actinb* and expressed as ratios to control data. Data are combined from six independent experiments. Statistical significance was evaluated by using one-way ANOVA for comparison of multiple groups and the Mann–Whitney test for two groups; ****p < 0.0001; ***p < 0.001; **p < 0.01; *p < 0.05; N.S. not significant.

In contrast to obesity-associated phenotypes, we found that the number of macrophages infiltrated into the epididymal adipose tissue was decreased by oral administration with αKetoA (Fig. 4a). Consistently, αKetoA decreased the expression level of the M1 macrophage marker *Nos2* and increased that of the M2 macrophage marker *Fizz1* in macrophages isolated from epididymal adipose tissues (Fig. 4b). In addition, in vitro assays using bone marrow-derived macrophages revealed that αKetoA influenced the polarization of M1 and M2 macrophages. Indeed, αKetoA decreased the expression levels of the M1 markers *Nos2* and *Cd86* yet promoted those of the M2 markers *Fizz1*, *Chi3l3*, and *Arg1* (Fig. 4c, d). The effects of αKetoA on the gene expression levels of *Nos2*, *Fizz1*, and *Arg1* were canceled by inhibition of PPARγ (Fig. 4c, d). These findings were consistent with a previous study showing the involvement of PPARγ in macrophage polarization to M2 phenotypes.^39^

In obesity-associated inflammation, adipocytes produce CCL2 and S100A8 for the recruitment of macrophages.^36–38,40^ Treatment with αKetoA had little effect on the expression of *Ccl2* and *S100a8* in adipocytes (Supplementary Fig. S6), suggesting that αKetoA acted directly on macrophages to inhibit their infiltration into adipose tissues and to alter macrophage polarization to M2 phenotypes.

**Fig. 5:**
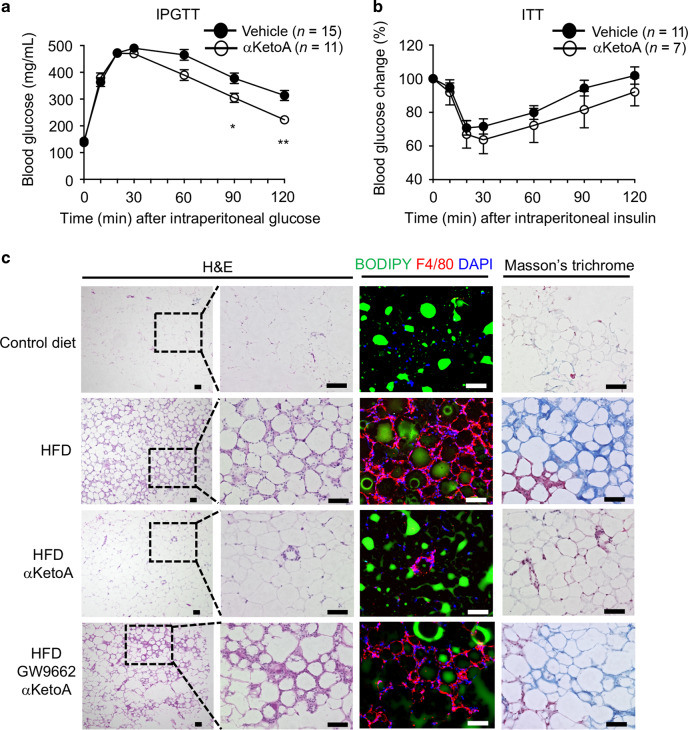
HFD-induced glucose intolerance is ameliorated by αKetoA through inhibiting adipose tissue inflammation. **a** IPGTT was performed after HFD feeding for 3 months with or without oral administration of αKetoA (dose: 10 μg/mouse, 3 times/week). **b** ITT was performed after HFD feeding for 3.5 months with or without oral administration of αKetoA (dose: 10 μg/mouse, 3 times/week). **c** After HFD feeding for 4 months with or without oral administration of αKetoA (dose: 10 μg/mouse, 3 times/week) and with or without intraperitoneal injection of GW9662, epididymal adipose tissues were examined histologically. Mice fed with control diet containing soybean oil were used as a control. Data are representative of four independent experiments (*n* = 12/group). Scale bars, 100 μm. Statistical significance was evaluated by using the Mann–Whitney test; **p < 0.01; *p < 0.05.

In accordance with these findings, intraperitoneal glucose tolerance test (IPGTT) and insulin tolerance test (ITT) revealed that αKetoA decreased HFD-induced glucose intolerance (Fig. 5a, b). We then examined HFD-induced adipose tissue remodeling, such as the development of crown-like structures and fibrosis, which play key roles in promoting chronic inflammation and metabolic disorders.^41^ Histologic analysis revealed that αKetoA ameliorated cellular infiltration into epididymal adipose tissues (Fig. 5c). Furthermore, immunohistochemical analysis using an anti-F4/80 mAb to visualize macrophages revealed that αKetoA inhibited the development of crown-like structures (Fig. 5c).

Chronic inflammation in adipose tissue eventually leads to the development of interstitial fibrosis, which causes adipose tissue dysfunction and ectopic lipid accumulation; these conditions subsequently lead to non-alcoholic steatohepatitis, which shows hepatic insulin resistance due to reduced expression of insulin receptor β.^42^ Consistent with the finding that αKetoA inhibited adipose tissue inflammation and decreased glucose intolerance, we found that αKetoA prevented the development of adipose tissue fibrosis, as evaluated by Masson’s trichrome staining (Fig. 5c). In line with our current findings regarding the contact hypersensitivity model, the activities of αKetoA in inhibiting the formation of crown-like structures and fibrosis were dependent on PPARγ because these activities were abrogated by treatment with GW9662 (Fig. 5c). These results collectively demonstrate that the αKetoA–PPARγ axis ameliorates HFD-induced adipose tissue remodeling without affecting obesity-associated increases in body weight and epididymal adipose tissues.

### Detection of αKetoA in human feces

Given that αKetoA showed anti-diabetic effects in mice, we next asked whether αKetoA levels are decreased in human diabetic patients. As it is generally known that ordinary intake of dietary ω3 oil is low in normal life, the fecal αKetoA levels were low and comparable between healthy people and diabetic patients (Supplementary Fig. S7a). To assess the correlation between α-linolenic acid and αKetoA in feces, we then established another cohort that included participants who consumed various amounts of dietary α-linolenic acid due to ad libitum intake of α-linolenic acid-rich linseed-related products. We found that the amount of αKetoA was positively correlated with that of α-linolenic acid (Supplementary Fig. S7b). Although the precise effects of αKetoA on human diabetes are a subject for future study, these findings collectively suggest that dietary intake of α-linolenic acid promotes the production of αKetoA in humans.

## Discussion

Accumulating evidence suggests that the intestinal microbiome influences host health and diseases, not only in the intestine but also in other tissues, including the respiratory tract, central nervous system, and skin, through the regulation of inflammation, allergy, and metabolic disorders.^43–46^ Microbial metabolites of food materials are known as ‘postbiotics’. Currently postbiotics are attracting attention as bioactive molecules that likely are important in the underlying mechanisms through which the intestinal microbiome can control multiple host organs remotely. Indeed, we detected αKetoA not only in feces but also in serum in mice. In addition, αKetoA exerted its anti-inflammatory activities through regulation of macrophage activities in the skin and adipose tissue to ameliorate contact hypersensitivity and metabolic disorder. A recent study similarly showed that the microbe-dependent ω6 linoleic acid metabolite HYA ameliorated metabolic disorders.^47^ HYA induced GPR40- and GPR120-dependent GLP1 secretion from enteroendocrine cells, facilitated glucose metabolism, and inhibited the development of obesity.^47^ Therefore, αKetoA and HYA are both microbe-dependent metabolites of essential fatty acids that improve glucose metabolism through the different molecular and cellular bases of the αKetoA–PPARγ axis in macrophages and the HYA–GPR40 and –GPR120 axes in enteroendocrine cells. These intestinal microbial metabolites, which are generated through reduction reactions, are chemically much more stable than the oxidation metabolites produced by the host, because the reduction metabolites lack the unstable conjugated double-bond structure. This stability enhances the usefulness of these intestinal microbial metabolites as postbiotics.

With current dietary habits, people tend to consume only low amounts of ω3 fatty acids. In agreement with this trend, our cohort study indicated that Japanese adults generally ingest small quantities of ω3 fatty acids, which resulted in barely detectable levels of αKetoA even in the feces of healthy participants. αKetoA could be increased to more than 1000 pg per 50 mg feces in humans when they consumed a diet rich in α-linolenic acid, thus suggesting that these levels would result in an anti-inflammatory effect. Although αKetoA is not a critical determinant in the development of diabetes, these findings suggest that increasing αKetoA levels through increased intake of α-linolenic acid might ameliorate diabetic inflammation in human patients. We plan to establish another cohort to directly evaluate the effect of dietary intake of α-linolenic acid and its metabolism to αKetoA in regard to the development of diabetes.

It is worth noting that the population having the same amount of α-linolenic acid contains both αKetoA-high and -low producers, indicating that the composition of the intestinal microbiota would affect the level of αKetoA. Conversion of α-linolenic acid to αHYA is potentially mediated by several types of bacteria, including *Lactobacillus plantarum*, *L. acidophilus*, *Streptococcus pyogens*, *Stenotrophomonas nitritireducens*, and *Flavobacterium* spp.,^11^ and that of αHYA to αKetoA is mediated by *L. plantarum* and *Flavobacterium* spp.^19,48^ Therefore, rather than dietary supplementation with precursor compounds, a better strategy might be to take αKetoA itself to obtain suitable anti-inflammatory effects, because the microbiota differs among people. Several fermented foods, including Japanese pickles, Korean kimchi, and German sauerkraut, are enriched with *L. plantarum*. Because these foods are produced through fermentation, they might contain bioactive microbial metabolites. Therefore, another prospective strategy involves adding the precursors of bioactive metabolites (e.g., α-linolenic acid) during fermentation to increase the amounts of desired bioactive microbial metabolites in food products.

In the contact hypersensitivity model, macrophages play important roles in the induction of iSALT formation by expressing CXCR2 ligands, which induces clustering of dermal dendritic cells.^34^ We found that αKetoA inhibited iSALT formation by decreasing the level of the inflammatory chemokine CXCL1. Consistently, we found that the nuclear translocation of NF-κB, which plays central roles in triggering inflammation by inducing the gene expression of pro-inflammatory cytokines and chemokines, including *Cxcl1,*^49^ was inhibited by αKetoA in macrophages in a PPARγ-dependent fashion. This scenario is in accordance with previous reports indicating that the activation of PPARγ suppresses NF-κB activation and consequent inflammatory responses.^50,51^

NF-κB-mediated gene induction of *Tnfa* and *Il1b* is a hallmark of M1 macrophage polarization.^52^ Consistent with the finding that the αKetoA–PPARγ axis inhibited NF-κB activity, αKetoA suppressed polarization to M1 macrophages. In addition, PPARγ activators are known to induce the polarization of macrophages to the M2 phenotype,^53,54^ thus supporting our finding that αKetoA promoted M2 macrophage polarization. αKetoA simultaneously inhibited M1 macrophage polarization and promoted M2 macrophage polarization, such that both activities contributed to the inhibition of adipose tissue inflammation. From the viewpoint of fibrosis, it is worth noting that macrophage production of nitric oxide plays a key role in the induction of adipose tissue fibrosis.^55^ Conversely, αKetoA reduced the gene expression of *Nos2* yet promoted that of *Arg1* in macrophages, thereby decreasing tissue levels of nitric oxide.

It is widely accepted that obesity is the critical determinant in inducing adipose tissue inflammation, which is the mechanism underlying the development of metabolic disorders.^36–38^ However, we found that αKetoA decreased glucose intolerance without affecting body weight gain, suggesting that obesity does not always lead to the development of metabolic disorders. The infiltration of macrophages is a primary event in obesity-induced adipose tissue inflammation.^36–38^ In obesity, adipocytes produce CCL2 and S100A8, which recruit CCR2-expressing pro-inflammatory M1 macrophages and monocytes to adipose tissue.^36–38,40^ We found that αKetoA did not alter the expression levels of *Ccl2* and *S100a8*; instead, αKetoA—in a PPARγ-dependent manner—prevented macrophages from infiltrating the adipose tissues of obese mice and inhibited fibrosis and the formation of crown-like structures. In addition to their effects on macrophage polarization, agonists of PPARγ (e.g., rosiglitazone and thiazolidinediones) suppress the chemotaxis of monocyte–macrophages.^56,57^ Macrophage-specific deletion of PPARγ increases the chemotactic response to CCL2.^58^ Mechanisms of PPARγ-mediated suppression of the chemotactic response include the down-regulation of CCR2 expression.^57,58^ Given that CCR2 expression is upregulated through NF-κB signaling in macrophages,^59^ PPARγ-mediated down-regulation of CCR2 might occur through the suppression of NF-κB activity. Together, these previous studies suggest that the αKetoA–PPARγ–NF-κB axis negatively regulates chemotaxis of pro-inflammatory M1 macrophages and monocytes into adipose tissue.

GW9662 used in this study is widely employed as a potent, irreversible, and selective PPARγ antagonist, which acts by covalently modifying a cysteine residue in the PPARγ ligand-binding domain.^60–63^ Indeed, GW9662 inhibits radioligand binding to PPARγ, PPARα, and PPARδ with pIC_50_s of 8.48 (IC_50_ = 3.3 nM), 7.49 (IC_50_ = 32 nM), and 5.69 (IC_50_ = 2000 nM), respectively. However, a recent study identified that GW9662 triggered gene expression via PPARδ, therefore, the off-target effect existed.^64^ Based on the fact, we should further address the specific role of PPARγ by using other methods, including genetically modified animals and gene knock-down system in a future study.

In summary, αKetoA is found as α-linolenic acid-derived postbiotics and as such is only extracted from dietary ω3 fatty acids in the presence of intestinal microbiota. We found that, by regulating various activities of macrophages, αKetoA exerted potent anti-inflammatory effects in mice and cynomolgus macaques, ameliorated skin inflammation, and decreased diabetic glucose intolerance. These results pave the way for the development of new drugs and probiotics, functional fermented foods, and postbiotics for the treatment of macrophage-associated inflammatory diseases, including skin inflammation and diabetes.

## Materials and Methods

### Animals

For lipidomics, female C57BL/6J wild-type mice (6 weeks old) were purchased from Japan SLC (Hamamatsu, Japan), and were maintained for 2 months on chemically defined diets containing 4% (wt/wt) dietary oil (soybean oil or linseed oil, Oriental Yeast, Tokyo, Japan)^65^ in the SPF animal facility at National Institutes of Biomedical Innovation, Health and Nutrition (NIBIOHN; Osaka, Japan). Male GF mice (ICR background), and their control ICR mice (age, 6 weeks) were purchased from Japan SLC (Hamamatsu, Japan); these mice were maintained for 2 months on chemically defined diets containing 4% (wt/wt) dietary oil (soybean oil or linseed oil) under GF or SPF conditions, respectively, at NIBIOHN and Oriental Bioservice, Inc. For contact hypersensitivity studies, female C57BL/6J wild-type mice (age, 7 weeks) were purchased from Japan SLC and maintained for 1 week before use in experiments in the SPF animal facility at NIBIOHN. These mice were maintained on a commercially available FR2 regular diet (Funabashi Farm, Chiba Japan). For diabetes experiments, male C57BL/6J wild-type mice (age, 8 weeks) were purchased from Japan SLC and CLEA Japan (Tokyo, Japan), and maintained in the SPF animal facility at NIBIOHN for 3–4 months on HFD composed of chemically defined materials.^65^ Mice were maintained under conditions (16:8 h light/dark cycle, 22–24 °C, and 50–60% humidity), with ad libitum access to food and distilled water. Mice were euthanized by cervical dislocation under isoflurane (Forane, AbbVie, North Chicago, Illinois, USA) anesthesia.

Male cynomolgus macaques (*Macaca fascicularis*; age, 2 years; weight; 2 kg) were maintained at the Tsukuba Primate Research Center, NIBIOHN, according to the “Rules for Animal Care and Management of Tsukuba Primate Center” and the “Guiding Principles for Animal Experiments using Non-human Primates” formulated by the Primate Society of Japan. All experiments were conducted in accordance with the guidelines of the Animal Care and Use Committee of NIBIOHN (DS25-2, DS26-41, DS27-47, DSR01-2, and DSR01-3). The study was carried out in compliance with the ARRIVE guidelines.

### LC-MS/MS

LC-MS/MS was performed as reported.^66^ Data analysis was performed by using the software Xcalibur 2.2 (ThermoFisher Scientific).

### Production of αHYA, αKetoA, and 13-oxo-cis-9,cis-15-octadecadienoic acid

To prepare αHYA and 13-hydroxy-*cis*-9,*cis*-15-octadecadienoic acid from α-linolenic acid, recombinant *E. coli* Rosetta2/pCLA-HY and Rosetta2(DE3)/pET21b-*fa-hy1* were used as catalysts, respectively.^28,67^ To prepare αKetoA and 13-oxo-*cis*-9,*cis*-15-octadecadienoic acid, recombinant *E. coli* Rosetta/pCLA-DH was used as the catalyst^29^ and purified αHYA and 13-hydroxy-*cis*-9,*cis*-15-octadecadienoic acid were used as substrates, respectively. These recombinants were cultivated in 10 mL Luria–Bertani medium at 37 °C for 12 h with shaking at 300 strokes/minute. Seed cultures were each transferred into 750 mL fresh Luria–Bertani medium and incubated at 37 °C for 2 h with shaking at 100 strokes/minute. After the addition of 1.0 mM isopropyl-β-thiogalactopyranoside, recombinants were incubated at 16 °C for 12 h with shaking at 100 strokes/minute. After incubation, recombinants were harvested by centrifugation and used as catalysts. The reaction conditions were as described previously.^28,29,67^ Reaction products were purified by using an Isolera One automated flash purification system equipped with a SNAP Ultra 10-g cartridge (Biotage, Stockholm, Sweden). The purity of the products exceeded 95%, according to gas chromatographic analysis.

### Induction of contact hypersensitivity in mice

Murine contact hypersensitivity was induced as described previously.^13^ In brief, on day 0, the abdominal skin of each mouse was shaved and then treated with 25 μL of 0.5% (vol/vol) DNFB (Nacalai Tesque, Kyoto, Japan) dissolved in a mixture of acetone and olive oil (acetone:olive oil, 4:1; both reagents from Nacalai Tesque). On day 5, the fronts and backs of both ears were challenged with 0.2% (vol/vol) DNFB (10 μL per site). In some experiments, mice intraperitoneally received either GW1100 (1 mg/kg body weight; Cayman Chemical, Ann Arbor, MI, USA), AH7614 (1 mg/kg body weight; Tocris Biosciences, Bristol, UK), or GW9662 (1 mg/kg body weight; Abcam plc, Cambridge, UK) which act as selective antagonist for GPR40, GPR120 or PPARγ, respectively.^60,68,69^ Ear thickness was measured by using a micrometer (model MDC-25MJ 293-230; Mitsutoyo, Kawasaki, Japan). Ear swelling was calculated as: (ear thickness [μm] after DNFB application) − (ear thickness [μm] before DNFB application) = Δ μm.

### Induction of contact hypersensitivity in cynomolgus macaques

Contact hypersensitivity in cynomolgus macaques was induced as described previously.^13^ αKetoA (1500 μg/350 μL) or 50% (vol/vol) ethanol dissolved in PBS (350 μL) as a vehicle control were applied topically to the right and left sides of the back, respectively, 30 min before DNFB challenge on days 5 and 7. αKetoA and vehicle control were additionally applied topically on days 6 and 11. Skin samples were obtained by means of biopsy on day 12.

### Induction of diabetes in mice

Mice were kept for 3–4 months on HFD composed of chemically defined materials in the SPF animal facility at NIBIOHN.^65^ During this period, mice were orally treated with αKetoA (dose, 10 μg/mouse) or 0.5% (vol/vol) ethanol dissolved in PBS as a vehicle control 3 times each week. In some experiments, mice intraperitoneally received GW9662 (1 mg/kg body weight) 30 min before oral administration of αKetoA.

### Cell isolation and flow cytometric analysis

Cell isolation and flow cytometry were performed as described previously.^13,40^ To avoid non-specific staining, cell samples were blocked with anti-CD16/32 monoclonal antibody (mAb) (dilution, 1:100; catalog no. 101320, TruStain fcX, BioLegend, San Diego, CA, USA). The following fluorescently labeled mAb were used for flowcytometric analysis: AF647–anti-I-A^b^ (1:100; 116412, BioLegend), APC-Cy7–anti-CD11b (1:100; 101226, BioLegend), PE-Cy7–anti-F4/80 (1:100; 123114, BioLegend), BV421–anti-CD11c (1:25; 117330, BioLegend), FITC–anti-Gr1 (1:100; 108406, BioLegend), APC-Cy7–anti-CD3ε (1:100; 557596, BD Biosciences), PE–anti-IFNγ (1:100; 505808, BioLegend), FITC–anti-TCRβ (1:100; 109206, BioLegend), and BV421–anti-CD45 (1:100; 103133, BioLegend). To stain intracellular cytokines, cells were treated for 60 min with brefeldin A (1:1000; 420601, BioLegend) during collagenase treatment. After being stained for viability and cell-surface markers, cells were fixed and permeabilized (Cytofix/Cytoperm Fixation/Permeabilization Kit, BD Biosciences) according to the manufacturer’s protocol. Dead cells were detected by using 7-AAD (1:100; 420404, BioLegend) or Zombie-NIR Fixable Viability Kit (1:100; 423106, BioLegend) and excluded from analysis. Flow cytometric analysis and cell isolation were conducted by using FACSAria (BD Biosciences). Data were analyzed by using FlowJo 9.9 (Tree Star, Ashland, OR, USA).

### Histologic analysis

Frozen and paraffin tissues were analyzed histologically as described previously.^13^ For staining of paraffin tissues with anti-CD3 mAb (Clone: CD3-12, GeneTex), antigen retrieval was conducted by heating sections in 1 mM EDTA solution (pH 9.0) for 15 min in a microwave oven after deparaffinization. The following antibodies and reagents were used for immunohistologic analysis: purified-anti-CD3ε mAb (1:100; 100302, for frozen tissue, BioLegend), purified-anti-I-A/I-E mAb (1:100: 107602, BioLegend), purified-anti-CD3 mAb (1:100; GTX42110, for paraffin tissue, GeneTex), purified-anti-F4/80 mAb (1:100; 123102, BioLegend), Cy3–anti-Armenian hamster IgG (1:200; 127-165-160, Jackson Immunoresearch Laboratories, West Grove, PA, USA), Cy3–anti-rat IgG (1:200; 712-165-153, Jackson Immunoresearch Laboratories), AF488–anti-rat IgG (1:200; A-11006, ThermoFisher Scientific), and BODIPY493/503 (1:1000; D3922, Molecular Probes, Eugene, OR, USA). Masson’s trichrome staining was conducted by using Trichrome Stain Kit (Modified Masson’s, ScyTek Laboratories, Logan, UT, USA), according to the manufacturer’s protocol. Tissue sections were examined under a fluorescence microscope (model BZ-9000, Keyence, Osaka, Japan).

### ELISA for CXCL1 and CXCL2

The amounts of CXCL1 and CXCL2 proteins in ear homogenates were analyzed by using Mouse CXCL1/KC Quantikine ELISA Kit (R&D Systems, Minneapolis, Minnesota, USA) and Mouse CXCL2 Quantikine ELISA Kit (R&D Systems), respectively, according to the manufacturer’s protocol. In brief, ear samples were homogenized for 30 sec with one 4.8-ϕ and three 3.2-ϕ beads in PBS containing protease inhibitor (P8340, Sigma) and centrifuged (10,000 rpm, 20 min, 4 °C); supernatants were collected and used for ELISA (protein concentration; 4 mg/mL). Absorbance at OD_450_ and OD_570_ was measured by using an iMark microplate reader (Bio-Rad, Hercules, CA, USA).

### Reverse transcription and quantitative PCR analysis

Reverse transcription and quantitative PCR analysis were performed as described.^13^ Primer sequences are as follows: *Cxcl1* sense, 5′-gactccagccacactccaac-3′; *Cxcl1* anti-sense, 5′-tgacagcgcagctcattg-3′; *Cxcl2* sense, 5′-aaaatcatccaaaagatactgaacaa-3′; *Cxcl2* anti-sense, 5′-ctttggttcttccgttgagg-3′; *Pparg* sense, 5′-gaaagacaacggacaaatcacc-3′; *Pparg* anti-sense, 5′-gggggtgatatgtttgaacttg-3′; *Nos2* sense, 5′-ctttgccacggacgagac-3′; *Nos2* anti-sense, 5′-tcattgtactctgagggctgac-3′; *Fizz1* sense, 5′-ccctccactgtaacgaagactc-3′; *Fizz1* anti-sense, 5′-cacacccagtagcagtcatcc-3′; *Chi3l3* sense, 5′-aagaacactgagctaaaaactctcct-3′; *Chi3l3* anti-sense, 5′-gagaccatggcactgaacg-3′; *Arg1* sense, 5′-gaatctgcatgggcaacc-3′; *Arg1* anti-sense, 5′-gaatcctggtacatctgggaac-3′; *Cd86* sense, 5′-gaagccgaatcagcctagc-3′; *Cd86* anti-sense, 5′-cagcgttactatcccgctct-3′; *Actinb* sense, 5′-aaggccaaccgtgaaaagat-3′; and *Actinb* anti-sense, 5′-gtggtacgaccagaggcatac-3′.

### In vitro assay of bone marrow-derived macrophages

Bone marrow cells were prepared from the femurs and tibias of 5- to 8-week-old C57BL/6J wild-type mice, and the differentiation of macrophages was induced as described previously^34^ with modification.

For immunocytochemistry, bone marrow cells were cultured on microscope cover glasses (18 mm; Matsunami, Osaka, Japan); placed in 12-well tissue culture plates (2 × 10^4^ cells/mL/well; Corning, Corning, NY, USA) containing Dulbecco’s modified Eagle medium (high glucose, Nacalai Tesque) supplemented with macrophage colony-stimulating factor (50 ng/mL; Peprotech, Cranbury, NJ, USA), 10% (vol/vol) fetal bovine serum (Gibco), and 1% (vol/vol) penicillin and streptomycin (Nacalai Tesque); and incubated at 37 °C in 5% CO_2_. Culture medium was replaced on days 3, 7, and 10. On day 7, cells were incubated with GW9662 (1 μM) or 0.1% (vol/vol) ethanol as a vehicle control for 30 min and incubated with interleukin (IL)-4 (20 ng/mL; Peprotech) and either αKetoA (30 nM) or 0.1% (vol/vol) ethanol as a vehicle control. On day 10, cells were incubated with GW9662 (1 μM) or 0.1% (vol/vol) ethanol as a vehicle control for 30 min and incubated with IL-1α (10 ng/mL; Peprotech) and either αKetoA (30 nM) or 0.1% (vol/vol) ethanol as a vehicle control for 30 min. Cells on the microplates were fixed with 4% (vol/vol) paraformaldehyde (Nacalai Tesque) for 20 min, washed with PBS, and then permeabilized with 0.5% (vol/vol) Triton X-100 (Nacalai Tesque) for 5 min. Samples were then washed with PBS and incubated with 2% (vol/vol) newborn calf serum for 30 min for blocking. Then, samples were stained with primary antibodies—anti-NF-κB p65 rabbit mAb (1:100; 8242, Cell Signaling Technology, Danvers, MA, USA) and purified anti-F4/80 rat mAb (1:100; 123102, BioLegend)—for 16 h at 4 °C. Samples were washed with PBS and then stained with secondary antibodies—AF488–anti-rabbit IgG (1:200; A-11034, ThermoFisher Scientific) and Cy3–anti-rat IgG (Jackson Immunoresearch Laboratories; 712-165-153; 1:200)—for 1 h at room temperature. Samples were then washed with PBS, stained with DAPI, and examined under a fluorescence microscope (model BZ-9000; Keyence).

For the analysis of chemokine expression, bone marrow cells were cultured in 6-cm dishes (1 × 10^5^ cells/mL, 5 mL/well; RepCell dishes, CellSeed, Tokyo, Japan) containing Dulbecco’s modified Eagle medium (high glucose) supplemented with macrophage colony-stimulating factor (50 ng/mL), 10% (vol/vol) fetal bovine serum, and 1% (vol/vol) penicillin and streptomycin at 37 °C and 5% CO_2_. Culture medium was replaced on days 3, 7, and 10. On day 7, cells were incubated with IL-4 (20 ng/mL) and either αKetoA (30 nM) or 0.1% (vol/vol) ethanol as a vehicle control. On day 10, cells were stimulated with IL-1α (10 ng/mL) with αKetoA (30 nM) or 0.1% (vol/vol) ethanol as a vehicle control. On day 11, mRNA was prepared from cells and used for reverse transcription and quantitative PCR analysis of the expression of *Cxcl1* and *Cxcl2*.

To analyze polarization of M1 and M2 macrophages, bone marrow cells were cultured in 6-cm dishes (1 × 10^5^ cells/mL, 5 mL/well; RepCell dishes, CellSeed) containing Dulbecco’s modified Eagle medium (high glucose) supplemented with macrophage colony-stimulating factor (50 ng/mL), 10% (vol/vol) fetal bovine serum, and 1% (vol/vol) penicillin and streptomycin at 37 °C and 5% CO_2_. Culture medium was replaced on days 3 and 5. On day 5, cells were stimulated with IFNγ (10 ng/mL; Peprotech) or IL-4 (20 ng/mL) to induce their differentiation to M1 and M2 macrophages, respectively.^34^ Cells were incubated with αKetoA (30 nM) or 0.1% (vol/vol) ethanol as a vehicle control for 30 min before cytokine stimulation. On day 7, mRNA samples were prepared and used for reverse transcription and quantitative PCR analysis of the expression of *Nos2* and *CD86* as M1 polarization markers and of *Fizz1*, *Arg1*, and *Chi3l3* as M2 polarization markers.

### TGFα-shedding assay

TGFα-shedding assays were performed as described previously.^70^ The agonistic activities of αKetoA, αHYA, and α-linolenic acid (3 μM) toward GPR40 and GPR120 were evaluated; 13-oxo-*cis*-9,*cis*-15-octadecadienoic acid was used as the positive control.^33^

### IPGTT and ITT

IPGTT and ITT were performed as described previously^71^ with modification. In brief, for IPGTT, mice were fasted overnight (16 h) and then injected intraperitoneally with d-(+)-glucose (20% solution; 2 g/kg body weight; Nacalai Tesque). For ITT, mice in the randomly fed state were injected intraperitoneally with human regular insulin (1.0 U/kg body weight; Eli Lilly, Indiana, USA). To measure blood glucose levels, blood was obtained from the tail veil by cutting with a single-edged blade (Feather, Osaka, Japan) and measured by using One Touch Ultra Vue (LifeScan Japan, Tokyo, Japan) before and after glucose injection at indicated time points.

### Human samples and ethics

Fecal samples were collected from participants in two human cohort studies. One includes healthy and diabetic patients who were recruited from Shunan City Shinnanyo Hospital (Shunan City, Yamaguchi, Japan) and surrounding communities; the other study involves healthy adult volunteers who were recruited from the communities around NIBIOHN (Ibaraki City, Osaka, Japan). All experiments were approved by the Ethics Committee of NIBIOHN (approval numbers: 177-07 and 154-10) and were conducted in accordance with their guidelines; informed consent was obtained from all participants. All samples were stored at −80 °C until use. All participants had no history of cancer, cardiovascular, liver, or gastrointestinal disease; candidates who took antibiotics, laxatives, or antiflatulents within a month before sample collection were excluded.

### Statistical analysis

Statistical significance was evaluated through one-way ANOVA for comparison of multiple groups and the Mann–Whitney test for two groups (Prism 6, GraphPad Software, La Jolla, CA, USA). A *P* value less than 0.05 was considered to be significant.

## Supplementary information

ARRIVE_guidelines

Revised_Supplementary_Figures
