# Supplementary material for: Intestinal microbe-dependent ω3 lipid metabolite αKetoA prevents inflammatory diseases in mice and cynomolgus macaques
Source: Mucosal Immunol. 2022 Jan 10;15(2):289–300. doi: 10.1038/s41385-021-00477-5 (PMC8866125; doi:10.1038/s41385-021-00477-5)
Supplement: Supplementary file 2 — Revised_Supplementary_Figures [file 41385_2021_477_MOESM2_ESM.pdf]

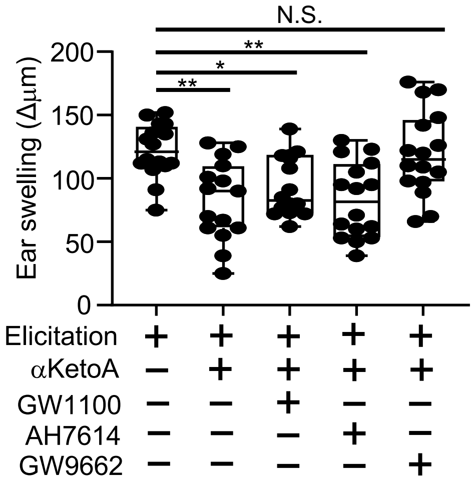

Supplementary Fig. S1 Contact hypersensitivity is ameliorated by αKetoA through a PPARγ-dependent manner. Mice were injected intraperitoneally with either GW1100, AH7614 or GW9662 30 minutes before oral administration of αKetoA on days 0 and 5. Mice were challenged with DNFB 90 minutes after the oral administration of αKetoA, and ear swelling was evaluated on day 7. Data are combined from 3 independent experiments and each point represents data from individual mice. Statistical significance was evaluated by using one-way ANOVA; ***p* < 0.01; **p* < 0.05; N.S., not significant.

Supplementary Fig. S1

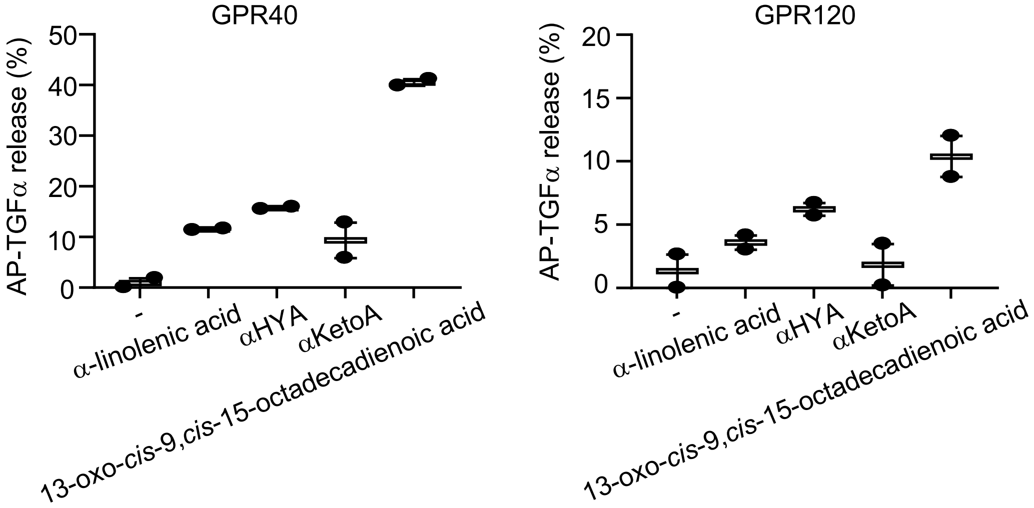

Supplementary Fig. S2 αKetoA has little agonistic activity to GPR40 and GPR120. TGFα-shedding assays were performed to evaluate the agonistic activities of the indicated fatty acids to GPR40 and GPR120; 13-oxo-*cis*-9,*cis*-15-octadecadienoic acid was used as a positive control. Data are representative of 3 independent experiments with similar results (duplicate assays).

Supplementary Fig. S2

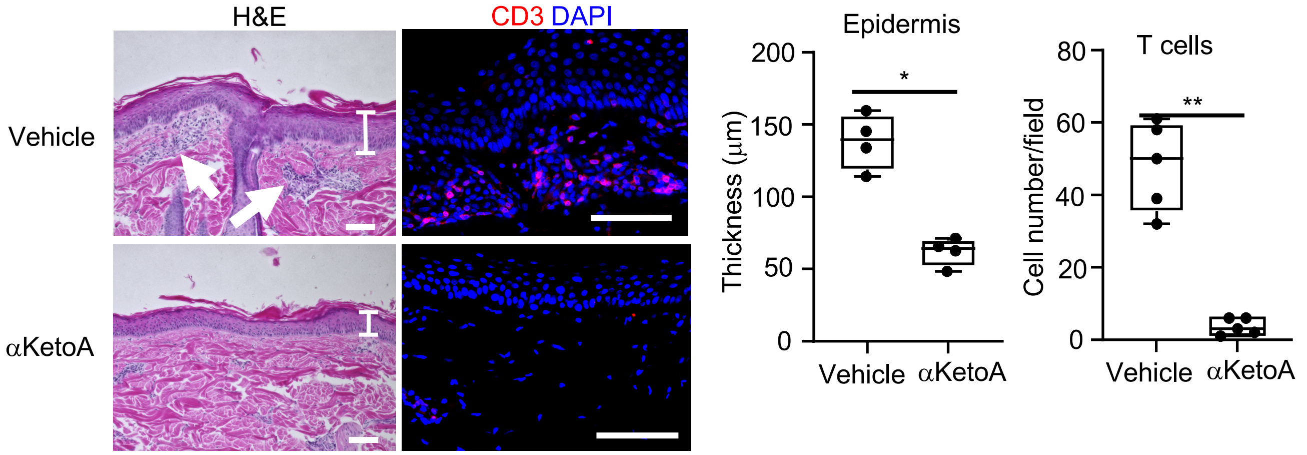

Supplementary Fig. S3 Contact hypersensitivity is ameliorated by αKetoA in cynomolgus macaques. DNFB-induced contact hypersensitivity was induced as described in the Methods. Skin biopsy samples were embedded in paraffin blocks, and resulting sections were analyzed by using hematoxylin & eosin staining or the indicated antibodies and reagent. Data are representative of 2 independent experiments (n = 2/group). Arrows indicate cell infiltration, and bars indicate thickening of the epidermis. Scale bars, 100 μm. Epidermal thickness and numbers of T cells were evaluated histologically (n = 4 or 5 sections/group), Statistical significance was evaluated by using the Mann–Whitney test; ***p* < 0.01; **p* < 0.05.

Supplementary Fig. S3

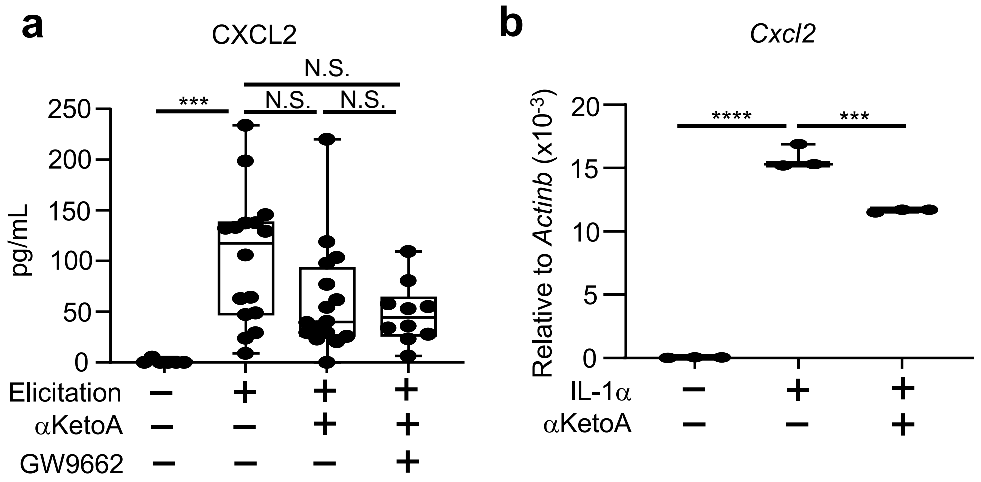

Supplementary Fig. S4 Effects of αKetoA on the expression of CXCL2. **a** Ear homogenates were prepared on days 6 and 7 of the contact hypersensitivity model and examined by ELISA to determine the amount of CXCL2. Data are combined from 5 independent experiments. **b** *In vitro* assay of bone marrow-derived macrophages. Bone marrow cells were incubated as described in the Methods section and stimulated with IL-1α with or without αKetoA to analyze the gene expression level of *Cxcl2*, which was normalized to that of *Actinb*. Data are representative of 3 independent experiments with similar results (triplicate assays). Statistical significance was evaluated by using one-way ANOVA; ****$p < 0.0001$; ***$p < 0.001$; N.S., not significant.

Supplementary Fig. S4

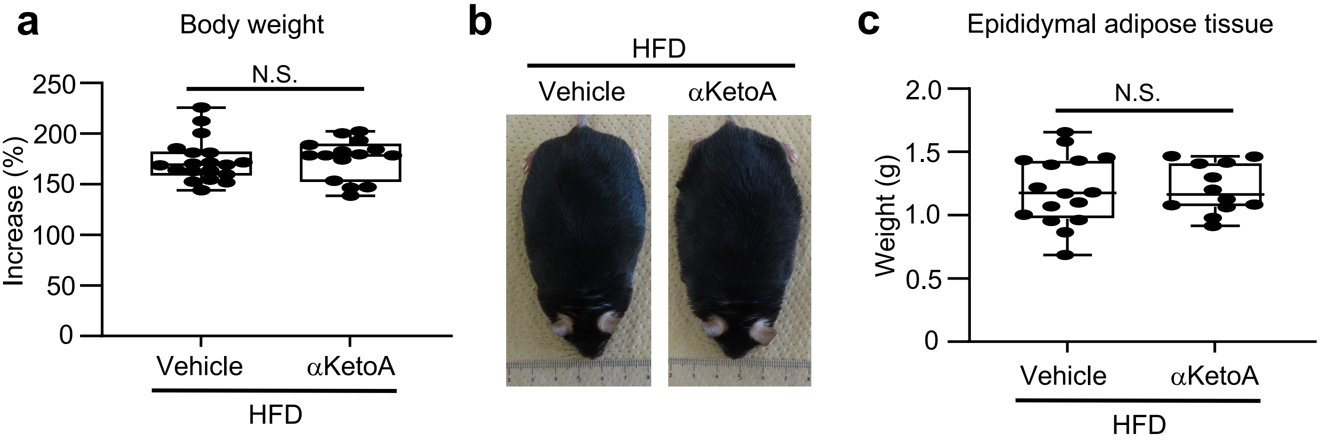

Supplementary Fig. S5 αKetoA has little effect on the development of HFD-induced obesity. **a** Mice were fed HFD for 3 months with or without oral administration of αKetoA (dose: 10 μg/mouse, 3 times/week) and their body weight measured. **b-c** Representative photograph of mice (b) and the weight of epididymal adipose tissues (c) after feeding of HFD for 4 months with or without oral administration of αKetoA (dose: 10 μg/mouse, 3 times/week). Data are combined from 4 independent experiments, and each point represents an individual mouse. Statistical significance was evaluated by using the Mann–Whitney test; N.S., not significant.

Supplementary Fig. S5

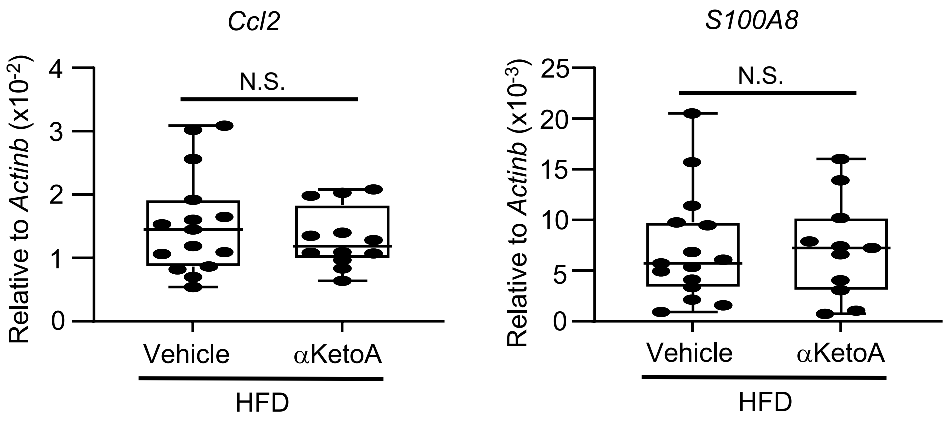

Supplementary Fig. S6 αKetoA has little effect on the gene expression levels of *Ccl2* and *S100A8*. After mice were fed a HFD for 4 months with or without oral administration of αKetoA (dose: 10 μg/mouse, 3 times/week), mature adipocytes were fractionated, and the gene expression levels of *Ccl2* and *S100a8* were examined by reverse transcription and quantitative PCR analysis. Data are combined from 4 independent experiments, and each point represents an individual mouse. Statistical significance was evaluated by using the Mann–Whitney test; N.S., not significant.

Supplementary Fig. S6

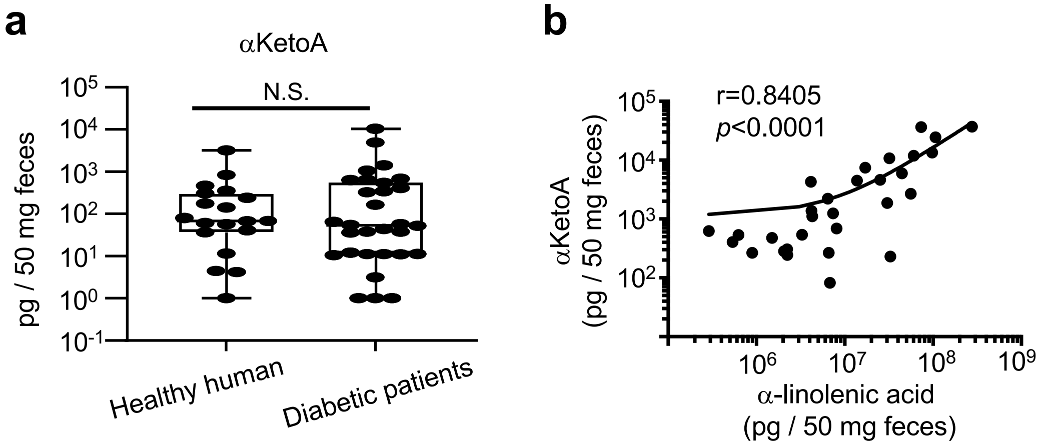

Supplementary Fig. S7 Detection of αKetoA in human feces. **a** Human fecal samples were collected from diabetic patients (n=30 [18 males, 12 females], age=58.5 ± 12.5 [range: 34-78]) and healthy adult volunteers (n=19 [14 males, 5 females], age=52.9 ± 10.9 [range: 34-75]), who were recruited from Shunan City Shinnanyo Hospital and the communities around the Shinnanyo Hospital at Shunan city, Yamaguchi, Japan. Feces were examined by LC-MS/MS for the detection of αKetoA, and their amounts were compared between healthy and diabetic patients. Statistical significance was evaluated by using the Mann–Whitney test; N.S., not significant. **b** Human fecal samples were collected from healthy adult volunteers (n=30 [10 males, 20 females], age=41.1 ± 10.7 [range: 25-63]) who were recruited from the communities around NIBIOHN at Ibaraki City, Osaka, Japan. Feces were examined by LC-MS/MS for the quantification of αKetoA and α-linolenic acid. Statistical correlation was evaluated by using Pearson *r*.

Supplementary Fig. S7
